# Trauma Research and Its Importance

**DOI:** 10.5812/atr.5287

**Published:** 2012-06-01

**Authors:** Esmaeil Fakharian

**Affiliations:** 1 Trauma Research Center, Kashan University of Medical Sciences, Kashan, IR Iran

**Keywords:** Accidents, Death, Wound and Injuries

From the time Abel was killed by his brother, Cain, in an assault action to his head as the first case of death on the earth (1), to the decapitation of the giant warrior Goliath by the young David after hurling a stone with his sling and hitting his head (2), to the death of many people in such natural disasters as earthquakes, like the excavated skeletons of a mother and her child in ancient Sialk Hills in Kashan-IR Iran related to more than 7000 years ago with the evidence of skull fracture in them, to the first automobile fatality of any kind in 1869 in which the scientist Mary Ward was thrown from her vehicle near a small town in County Offaly, Ireland (3), to the death of 44 years old Bridget Driscoll on August 17, 1896 when crossing the grounds of the Crystal Palace in London, as the first pedestrian victim , killed in an automobile accident (3), to the death of Pierre Curie, the husband of Marie Curie and Noble Laureate of physics, on April 19, 1906 in an accident by a heavy horse drawn cart and running over of the wheels over his head and fracturing his skull (4, 5), to the death of Gholam Hossein Darvish (6), famous Persian classical musician at age of 54 on November 22, 1926 in an accident hitting his carriage by a lorry resulting in his immediate death, as the first case of car accident death in Iran, the number of people dying for many different types of trauma has ever been growing and this has particularly increased after the introduction of motor vehicles and increasing speed in transportation (6). Latter on the severity and even the quality of injuries have been changed due to the effect of acceleration and deceleration on body organs. According to 2007 statistics from the National Highway Traffic Safety Association (NHTSA), motor vehicle accidents caused 41,059 fatalities on United States roadways, or nearly 14 deaths per 100,000 people (3). It has been reported that more than 20,000 people are killed in road traffic accidents in recent years in Iran. Considering the statement of the officer who recorded Bridget Driscoll’s accident scene “I trust that this sort of nonsense will never happen again” (3), Could estimate the depth of the changes happened in our life style in the past century; to which it seems that man has had no definite concept in advance. All of these are part of the numbers of injuries and does not include cases of falling down as another major cause of injury; occupational, sport and house hold events to name some. However, there has also been significant progress in different fields of medicine leading improvement in therapeutic strategies and its related armamentarium for trauma. In spite of these improvements, the need of further studies and scrutinizing different aspects of trauma and injuries still exists. 

Trauma Research Center of Kashan University of Medical Sciences (KAUMS) in its own way for contribution to the advancement of trauma in all aspects of prevention, treatment and rehabilitation is going to facilitate sharing of data between the scientists and authorities of this field, and so has published its new journal and invites all national, regional and abroad specialists to submit their experiences and researches for publishing in the “Archives of Trauma Research”. 

We hope to arrange for a better style of life with fewer deaths due to any kind of trauma. For this we think globally, and act locally.
